# Effects of 8-Week Creatine Supplementation With and Without Ubiquinol on Sperm Creatine Metabolism Biomarkers

**DOI:** 10.1080/15502783.2025.2533663

**Published:** 2025-08-01

**Authors:** David Nedeljkovic, Nikola Todorovic, Bogdan Andjelic, Sergej M. Ostojic

**Affiliations:** a Applied Bioenergetics Lab, Faculty of Sport and Physical Education, University of Novi Sad, Novi Sad, Serbia; b Sport and Exercise Sciences Research Unit, University of Palermo, Palermo, Italy; c Department of Nutrition and Public Health, University of Agder, Kristiansand, Norway; d Faculty of Health Sciences, University of Pecs, Pecs, Hungary

**Keywords:** Creatine supplementation, spermatozoa, ubiquinol, creatine kinase, biomarkers, dietary supplements

## Abstract

**Background:**

Rising interest in creatine’s role in sperm energy metabolism highlights its potential to enhance male fertility, with semen biomarkers providing insights into its impact on sperm function and fertility.

**Methods:**

Fifteen volunteers (mean age 25.0 ± 6.1 years; BMI 25.1 ± 2.0 kg/m^2^) were randomly assigned to one of three groups: Group 1 received received 5 g of creatine monohydrate plus 200 mg of ubiquinol, Group 2 received 5 g of creatine monohydrate, and the control group received 5 g of inulin daily for 8 weeks. Semen samples were collected after 3–5 days of abstinence and analyzed for creatine and creatine kinase (CK) levels.

**Results:**

The sperm CK levels showed a non-significant increase in the creatine-ubiquinol group, from 257.5 ± 140.6 IU/L at baseline to 455.2 ± 249.2 IU/L at 8-week follow up (*P* = 0.07; Cohen’s *d* = 0.88). Sperm creatine concentration also increased non-significantly, from 455.2 ± 188.9 µmol/L to 570.7 ± 228.1 µmol/L (*P* = 0.06; Cohen’s *d* = 0.55). In the creatine-only group, sperm CK decreased from 454.3 ± 106.5 IU/L to 422.9 ± 193.7 IU/L (*P* = 0.58; Cohen’s *d* = 0.20), while sperm creatine increased slightly from 499.4 ± 180.7 µmol/L to 564.3 ± 284.4 µmol/L (*P* = 0.71; Cohen’s *d* = 0.27). In the control group, both sperm CK and creatine concentrations decreased. CK dropped from 462.1 ± 229.1 IU/L to 419.9 ± 248.5 IU/L (*P* = 0.76; Cohen’s *d* = 0.17), and creatine declined from 464.8 ± 205.1 µmol/L to 459.5 ± 236.2 µmol/L (*P* = 0.90; Cohen’s *d* = 0.02). Friedman’s two-way ANOVA by ranks indicated a significant interaction effect for both sperm creatine and CK concentrations between the intervention groups (*P* < 0.05).

**Conclusion:**

Creatine, both alone and in combination with ubiquinol, led to increases in sperm creatine and CK concentrations, while these biomarkers remained unchanged in the control group. These results highlight the potential metabolic effects of creatine supplementation, warranting further investigation.

